# Technology Selection for Inline Topography Measurement with Rover-Borne Laser Spectrometers

**DOI:** 10.3390/s24092872

**Published:** 2024-04-30

**Authors:** Conor Ryan, Tobias Haist, Gennadii Laskin, Susanne Schröder, Stephan Reichelt

**Affiliations:** 1 Institute for Applied Optics (ITO), University of Stuttgart, 70569 Stuttgart, Germany; 2 Institute of Optical Sensor Systems, German Aerospace Center, 12489 Berlin, Germany; 3 Department of Production Control, Fraunhofer Institute for Physical Measurement Techniques IPM, 79110 Freiburg, Germany

**Keywords:** topography, 3D imaging, laser spectroscopy, space exploration, rover payload, Raman spectroscopy, LIBS

## Abstract

This work studies enhancing the capabilities of compact laser spectroscopes integrated into space-exploration rovers by adding 3D topography measurement techniques. Laser spectroscopy enables the in situ analysis of sample composition, aiding in the understanding of the geological history of extraterrestrial bodies. To complement spectroscopic data, the inclusion of 3D imaging is proposed to provide unprecedented contextual information. The morphological information aids material characterization and hence the constraining of rock and mineral histories. Assigning height information to lateral pixels creates topographies, which offer a more complete spatial dataset than contextual 2D imaging. To aid the integration of 3D measurement into future proposals for rover-based laser spectrometers, the relevant scientific, rover, and sample constraints are outlined. The candidate 3D technologies are discussed, and estimates of performance, weight, and power consumptions guide the down-selection process in three application examples. Technology choice is discussed from different perspectives. Inline microscopic fringe-projection profilometry, incoherent digital holography, and multiwavelength digital holography are found to be promising candidates for further development.

## 1. Introduction

Laser spectroscopic techniques such as Raman spectroscopy and laser-induced breakdown spectroscopy (LIBS) are increasingly used for in situ, geomaterial analysis on Earth and in extraterrestrial applications. Integrated in space-exploration rovers, these instruments provide data about the chemical and molecular properties of rocks and soils that allow scientists to infer past and present processes on extraterrestrial bodies [1,2].

To complement spectroscopic data, imaging systems may add contextual information to spectra in two ways. Large-field-of-view cameras may be used to locate/plan measurements within the immediate environment, such as the use of navigation images of the *Curiosity* rover to plan the spectral measurements of the ChemCam instrument [2]. Alternatively, imaging may provide finely resolved spatial information at sub-millimeter resolution. Such imagers may be in line with laser spectroscopes, such as the remote micro-imager (RMI) in the SuperCam instrument [3], or may be independent “hand lens imagers” positioned/steered by robotics, such as Watson [4] and CLUPI [5].

The spatial content of rocks and minerals is their morphology, which is affected by erosion, cooling rates, volcanic activity, meteoric bombardment, chemical activity and solar radiation exposure. The morphological manifestation of these processes are textures, grain sizes, shapes, cracks and other micro-features whose measurement constrains the set of possible histories [5,6], provide ground truth for estimated spatial parameters from remote sensing [7,8] and is combined with information from other sources into multiscale datasets [9]. As input for the science of regolith mechanics, they could support rover or lander operations as well as planning for in situ resource utilization. Microscale spatial information has so far been accessed using 2D cameras by the aforementioned context imagers and hand lens imagers.

However, due to the projection inherent in 2D imaging, access to morphology is restricted. Accuracy and utility of estimated 3D properties (e.g., grain edge roundness) from single 2D images is limited [10]. For samples returned to terrestrial laboratories, thin-section preparations are characterized with 2D imaging [11], or X-ray computed micro-tomography [12] or scanning electron microscopy [12,13] is used for 3D analysis. Such techniques are not reducible to simple add-ons to VIS/NIR laser spectroscopes.

**Figure 1:**
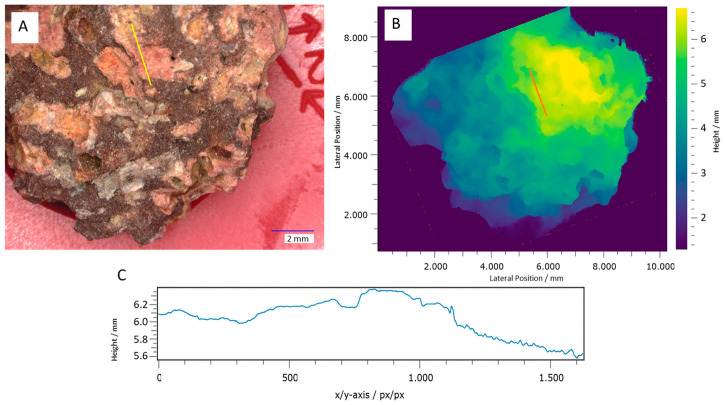
(**A**) A multispectral 2D image of an aged basalt sample, captured with a microscope. (**B**) The topography measurement of the same sample, measured with multiwavelength digital holography, revealing (**C**) locally varying roughness and an angular profile. The line direction is from bottom right (x/*y*-axis pixel 0) to top left x/*y*-axis pixel 1630. The topography of the aged basalt sample was measured using multiwavelength digital holography (Section 3.8).

If height information can be assigned to each lateral pixel in a 2D image, the morphology of the imaged surface, called the topography, is created. Topographies contain a fuller subset of spatial information than images, providing geologists with more complete and accurate data. This is exemplified by the topography in Figure 1.

Three-dimensional information has been generated by rovers from combinations of 2D images from several cameras or camera poses, but other approaches may produce better results with less constraints. Photogrammetric 3D (Section 3.3) has been implemented with two cameras in one stereo instrument, such as MastCam-Z [14] aboard *Perseverance* and PanCam [15] aboard *Rosalind Franklin*, with depth resolutions in the range of a few to a few tens of millimeters. Motion of a single camera (MAHLI on *Curiosity*) has been shown to allow depth resolution to a few hundred micrometers [16]. Combinations of multiple *Perseverance* cameras [9] are used to yield 3D measurements, although the resulting depth performance information is unavailable. Fringe projection approaches (Section 3.4) have been used to determine object distance to within 500 µm for PIXL [17] on *Perseverance*. Lingenauber et al. [18] suggested the use of plenoptic cameras (Appendix D) for rover-based 3D measurement and experimentally determined an RMS depth uncertainty of approximately 150 µm. Whilst sharing the property that achieved depth resolution is far worse than lateral resolution, the aforementioned examples are mostly not spectrometer-inline and have differing scientific goals and working distances.

Many other topography measurement techniques with compatible spectral ranges and optical architectures to laser spectroscopes exist. This work aims to compare topography measurement techniques based on the scientific and system requirements of a compact, rover-based laser spectrometer to help readers integrate 3D contextual measurement into their own future instrument proposals.

To this aim, requirements based on a heritage laser spectrometer and current rover trends are proposed in Section 2. A reference optical architecture for a rover spectrometer is scaled to three application scenarios, providing constraints for inline 3D measurement. In Section 3, candidate 3D technologies with qualitative and quantitative limits are summarized. In Section 4, estimations of spatial measurement performance, mass and power are presented and technology choice for inline topography measurement for rover-borne laser spectrometers is discussed.

## 2. Requirements for A Topographic Measurement Device

An optical technology is to be selected for a compact topography-measuring add-on for a laser-based spectrometer. The goal of the combined instrument is the collection of spectral and spatial information that provide clues about the geological/mineralogical identity and history of in situ extraterrestrial objects. In situ multispectral imaging aids mineral identification [5,6,19], so the topography add-on shall produce a multispectral depth map. If possible, NIR spectral bands should be used here for improved mineral differentiation. In the following, we attempt to refine the requirements for the topography measurement proposed in [18].

Since 2020, rovers below 100 kg have been more commonly planned, so the goal should be instrument accommodation in a <100 kg rover or lander. Small rovers accommodate the entire laser spectrometer internally, with viewports:

1. Outward facing, on a side or front panel [19].
2. Downward facing, on a bottom panel [20].
3. In or near contact with the sample [21].

The topography technology should be scalable to each of these three mounting configurations for flexibility in future mission scenarios. Regolith, or loose geological particles, are classified by length scales over five orders of magnitude. State-of-the-art imagers used for highly resolved in situ geological samples such as MAHLI [6] and CLUPI [5] have best-case sampling below 15 µm/pixel to allow distinction between sand and silt, stating this as a requirement for sedimentary, igneous and regolith geology. Fine resolution is important to validate microanalysis of return sample measurements, which may be biased to limited size scales [22] or altered by the sampling and atmospheric decent. The lateral as well as axial resolution for front-mounting shall be less than 30 µm at the object surface to make possible the resolving of fine sand. The finer the better, so bottom-mounting and contact-window cases should be 10 µm and 5 µm, respectively. For an instrument concept adaptable to any of the possible viewports, the technology should scale to measure topography in the configurations listed in Table 1 (derived in Appendix B).

The instrument should be compatible with external scanning optics, but not need it to function. To reduce potential scanner mass, the topography and spectral measurements shall be inline. A fixed focal length objective scanning the required working distance range is sensible for small ranges/objectives, but would be too voluminous for the front panel-mounted configuration. The autofocusing objective in this case must be a stationary, variable focus objective. Topography measurement should be compatible with both types of focusing. To reduce systems impact, the topography add-on should not increase the size, weight or power of the host spectrometer by more than 30%.

Space applications rule out certain technological elements. In this project, actuators with relative motion between contacting surfaces or precision requirements should be avoided. Only active optics with space heritage, low mass and simple electrical requirements should be used. Examples are transmission- or reflection-mode liquid crystal devices, acousto-optic modulators driven at fixed frequencies, or tribology-free piezoelectric translators. Additional illumination should be limited to LEDs or diode lasers.

Strong local slope variation is expected for in situ topography measurement, so robustness against surface slopes and highly 3D objects should be prioritized. For scientific evaluation, at least 90% of the measured sample points should yield valid data (depth) points.

A plausible option for in situ calibration of the topography measurement should be possible to ensure measurement accuracy while and after being subject to extreme conditions. Measurement time should be less than 1 h. Final data product shall be less than 1 GB, before compression.

Raman spectrometer sensitivity must be high (see [23] for more details), implying that the focusing objectives numerical aperture (NA) should remain above NA = 0.125 and shall not be telecentric (see Appendix A). While this is a reduction from the NA of 0.2 used in the RAX [24] and RLS [25] spectrometers, RAX’s signal-to-noise ratio was excellent when characterized on real objects and it can still be improved by a number of hardware and software means. Nonetheless, topography measurement hardware shall not reduce transmission in the spectroscope. Reducing the NA importantly allows a compact realization of increased working distance and inline scanning. Rover-borne Raman spectroscopy with much smaller collection NA exists using intensified time-gated detection [26], but mass and Raman spectrum quality require improvement. The sensitivity implies local night measurement, which can be exploited by topography measurement too.

For reference, assuming 532 nm light, a numerical aperture (NA) over 0.065 is needed to optically resolve any coarse silt grains (20–63 µm according to ISO classification [27]). Raman spectroscopy requirements drive NA more than spatial resolution requirements.

Raman and laser-induced breakdown spectroscopy (LIBS) (see [28] for more details) require autofocusing. For topographic measurement, axial scanning may be used, but any incurred magnification changes must be supported. Lastly, the spectrometer laser spot on the object need not be well resolved, but the targeted grain should be resolved. With the application requirements now outlined, we summarize potentially suitable 3D technologies and their limitations.

## 3. Relevant 2D and 3D Technologies

An overwhelming number of optical topographic techniques exist. The underlying physics, modern “workarounds” and expected performance are outlined here to aid selection. Performance depends on many factors, so estimates here are to be understood as such. As a shared system in several 3D techniques, an inline 2D imager add-on is first presented. Then, limits in depth measurement are outlined before the candidate 3D technologies are explained. Preclusion of several 3D techniques is discussed in Appendix D.

### 3.1. Baseline 2D Imager Add-On

**Figure 2:**
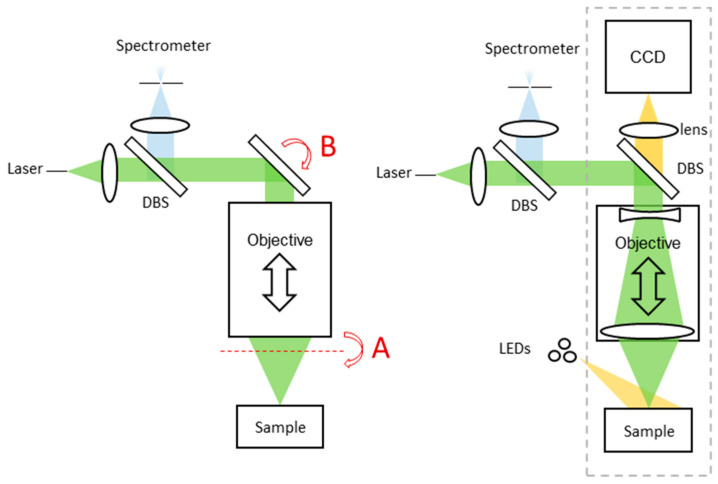
(**Left**) Simplified laser spectrometer architecture based on the RAX Raman spectrometer showing possible scanner locations A and B. (**Right**) Baseline implementation of an inline multispectral imaging camera, with the dashed line enclosing the imaging optical system of Figure A1.

Diffraction-based spectrometers are the focus of this work as they offer high resolving power without active components. A spectrometer architecture, applicable to Raman spectroscopy or LIBS, shown on the left in Figure 2 below, is based on the RAX Raman spectrometer [29]. Laser light is launched from a fiber whose tip is confocal with the spectrometer slit and illumination spot on the sample. Collimated beams traverse the instrument. An autofocusing objective is shared for laser excitation and detection. This objective has a large aperture for instrument sensitivity, but a small output beam diameter for miniaturization. A fixed focal length objective is axially translated. After collection, returning light is spectrally separated by a dichroic beam splitter (DBS) and sent to the spectrometer.

Topographic imaging techniques may utilize a baseline multispectral 2D imager, the concept of which is shown on the right in Figure 2. It uses a DBS for inline measurement and a lens to focus an image on the detector. Multispectral illumination is provided by external LEDs, allowing multispectral imagery without a resolution sacrifice. Measurement quality can be improved against steeply sloped and/or specular surfaces by using an LED ring for multidirectional illumination. A simplified three-lens optical model of the baseline imager is given in Appendix A.

Raman and LIBS emissions do not typically maintain polarization, so only spectral beam splitting can be inline without impairing the spectroscopy signal. This means topography measurement techniques using the existing spectrometer must use off-axis illumination, or trade illumination for spectroscope transmission. Techniques not exploiting the spectrometer should use a different spectral range.

Equations of microscopic imaging are included here as they are frequently referenced in this work and used in performance estimation. Given below are the lateral magnification M of an imager, the Rayleigh-defined lateral *δx* and axial *δz* resolution limits [30], geometric depth-of-field *DoF* and lateral object-field extent Δ*x* equations:

(1) $$M=\frac{NA_{in}}{NA_{out}}$$

(2) $$\delta x=\frac{0.61\lambda }{NA_{in}}$$

(3) $$\delta z_{Rayleigh}=\frac{2\lambda }{ΝA^{2}}$$

(4) $$DoF=\frac{NA_{out}c}{NA_{in}^{2}}$$

(5) $$\Delta x=\frac{L_{det}}{\left|M\right|}$$

where *NA_in_*, *NA_out_* are the numerical apertures at the object and detector sides respectively, *λ* is the mean wavelength of light, *c* is the allowable “circle of confusion” and *L_det_* is the detector side length. The factor 0.61 in Equation (2) becomes 0.82 for coherent light [30].

The spectrometer autofocus mechanism allows imaging at object planes outside the initial DoF. These images can be merged into a “focal stack” for all-in-focus images, increasing depth measurement range. The components needed for the multispectral baseline imaging add-on are a monochrome detector with objective, a dichroic beam splitter and multiple LEDs of different colors.

### 3.2. Depth Uncertainty in 3D Optical Measurement

**Figure 3:**
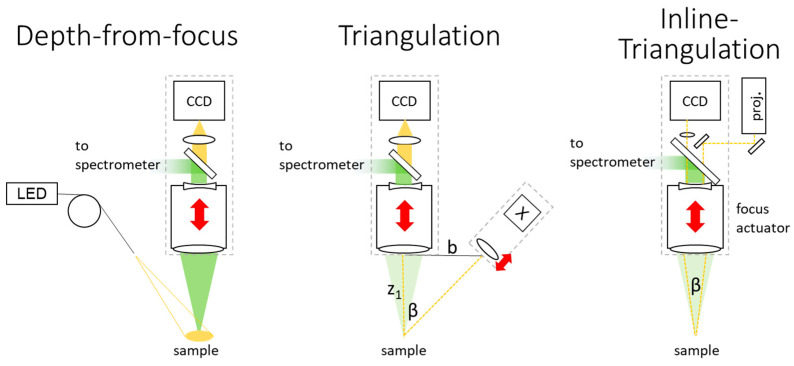
Exemplary configurations for depth from focus, off-axis triangulation and inline triangulation configurations for inline laser spectroscopy.

Three-dimensional measurement techniques can be classified by their depth uncertainty limits. Techniques discussed in this paper that may be regarded as variants of triangulation are depth from focus, confocal approaches, stereography, plenoptic imaging and fringe projection profilometry. Example architectures are given in Figure 3.

Triangulation is limited by speckle, which limits accuracy in determining the true location of a point on the object surface. An estimate of the uncertainty is given in [31]:

(6) $$\varepsilon_{z}=\frac{C\lambda }{2\pi NA_{in}sin(\beta )}$$

where *C* is the speckle contrast. If a single optical axis is used (i.e., depth from focus), the denominator becomes $2\pi NA_{in}^{2}$. From Equation (10), it follows that for triangulation, miniaturization worsens depth uncertainty.

Unlike triangulation, rough-surface interferometry techniques (coherence scanning interferometry and multiwavelength digital holography) can achieve a depth uncertainty limited to roughness within a resolution cell at the surface [31], which can decouple depth uncertainty from *NA_in_*.

### 3.3. Photogrammetry

In photogrammetry, measurements are derived from images. Stereography is a specific instance where two in-focus images from different perspectives are rectified and used for triangulation. The depth is encoded in the image coordinates disparities between jointly identifiable image points (homologous points). Increasing the perspective difference between the two cameras improves the depth resolution, but impairs the identification of homologous points. Stereography is common for rover navigation, but its main function there is hazard avoidance, which only requires coarse depth resolution. For this reason, stereoscopic rock metrology from the Mars rover *Spirit*, applied retrospectively in 2022, achieved depth resolution of only a few millimeters [32].

Low-texture surfaces provide few homologous points. Depth for every image pixel can be inferred from neighboring pixels with semi-global matching [33]. Alternatively, “active stereovision” applies structured projection to add surface features.

Stereography is capable of single-shot multispectral 3D evaluation using a Bayer mask on the camera(s). Miniaturization to a single detector design is possible by splitting the aperture of the camera, though with higher depth uncertainty. Alternatively, structure from motion (SfM) (see Appendix D) uses the motion of one camera to obtain multiple perspectives of a stationary sample, but the depth uncertainty is insufficient for this application.

Stereography lateral resolution may be given by Equation (2). Depth resolution is limited by disparity uncertainty *s_x_* between images. If the two cameras are separated by a distance *b*, depth resolution can be estimated as [34]:

(7) $$\delta z=\frac{z_{1}}{bM}s_{x}$$

While algorithms like SGM can determine disparities to sub-pixel accuracy [33] under the right conditions, we assume the uncertainty of disparity is the larger of 1 pixel or the Airy spot diameter at the detector.

The intersection of the field of view and *DoF* of both cameras provides an estimate of the measurement volume. The intersection is strongly limited by *DoF* if no refocusing is available. Thus, depth range per capture Δ*z* is the *DoF* and the lateral extent is reduced from Equation (5) to less than:

(8) $$\Delta x=\frac{DoF}{\sin\left(\beta \right)}$$

Outside this range, measurement uncertainty increases with defocus, quickly becoming a problem where microscopic resolution is needed. Stereography requires the baseline imager plus another refocusing camera as well as an active illumination system consisting of, e.g., a diffractive optical element and laser diode.

### 3.4. Fringe Projection Profilometry

Fringe projection profilometry (FPP) triangulates with a camera and an off-axis projector. A pattern is projected onto the object surface, the image of which from a different perspective is modulated by object height. The phase of the pattern is calculated over the image, unwrapped and transformed from image space to object space. Each step in the process has many variants, presented in an overview in [35]. FPP configurations for microscopy have been reviewed in [36].

Three categories of pattern are prevalent. Random pattern (e.g., speckles) projection allows correspondence of an area of the captured image to the corresponding unique area of the pattern. Projection can be compactly realized, but lateral sampling density is low and this single-shot technique is sensitive to noise and strong object inhomogeneities. Binary fringe projection is a multi-shot approach that improves robustness and lateral sampling density by unambiguously creating correspondence between projection and imaging using projection-field-dependent binary words. Sampling density and depth resolution depend on the finest projected period, and thus are NA-limited.

Phase-shifting FPP (PS-FPP) achieves the finest depth resolution, because the phase localization accuracy of every camera pixel can be orders of magnitude finer than the projection sinusoidal period. Therefore, periods can be coarser and optics smaller while independent depth values are still assigned to every pixel. Redundant phase-shifted captures increase the phase accuracy by averaging of errors, so more than three captures is common. A single-shot sinusoidal FPP method based on the Fourier transform of the image exists, but it struggles with object discontinuities and strong texture variation. For the above reasons, only PS-FPP is considered further.

PS-FPP is sensitive to changes in environmental lighting, but this should be negligible in local night measurements and due to the high NA objective. As with stereography, occlusions (shadowing) are an issue and a Bayer mask in the camera enables multispectral depth maps. Errors in the projected sinusoid profile cause errors in algorithms for phase shifting [37] and contrast limits performance. Multiple pattern periods are projected sequentially or in parallel with spectral multiplexing to eliminate fringe phase ambiguity.

Projection of interferometrically generated patterns allow miniature setups based on optical fiber tips [38,39] or Michelson interferometers [40]. These setups allow much larger measurement volumes and various means of phase shifting and period variation, albeit with increased speckle uncertainty.

The lateral resolution of PS-FPP follows Equation (2). The height *z* of an object point in the camera’s focal plane is linearly related to phase by $z={Τ}_{p}\Phi /2\pi sin\beta$, and hence the depth uncertainty can be approximated from the derivative of the phase–height relationship as:

(9) $$\delta z=\frac{T_{p}}{sin\beta }\left(\frac{\delta \Phi }{2\pi }\right)$$

where *δϕ*/2*π* is the phase uncertainty and *Τ_p_* the projected period of fringes at the object. PS-FPP allows phase estimation uncertainty to be 1/100 of a period [34], so in practice the depth uncertainty may be more limited by calibration and algorithm errors or speckle uncertainty. The speckle-limited depth uncertainty for the inline setup (Figure 3) can be calculated with Equation (10). For example, if *NA* = 0.125 is divided equally into two, that the maximum aperture angle and triangulation angles are 2*NA* = *β* = 0.125 and assuming *C* = 0.2, *λ* = 0.7 µm yields a depth uncertainty of 2.85 µm.

The measurement volume (Δ*x* and Δ*z*) is approximately bounded by the *FOV* and *DoF* of the imager. Defocus reduces the contrast of the sinusoidal pattern, so extended measurement volume can be achieved at the costs of reduced SNR and resolution. For fine resolution and a large depth-range measurement, refocusing projection optics are required. Inline FPP avoids this by making joint use of the spectrometer’s autofocus objective.

Inline PS-FPP with incoherent illumination requires the baseline imager as well as an inline-projection subsystem containing an LED, mirror, a lens and an SLM. AOMs [41], DMDs and LCDs [42] are rover-relevant options for the SLM.

### 3.5. Depth from Focus

Depth from focus (DFF) uses a series of images with incrementally shifted focal planes. The levels of defocus are calculated in subsections of each image. The best focus plane for each subsection is regarded as the axial position of the object surface.

As the best focus is evaluated based on the spatial distribution of intensity (image sharpness, image entropy, etc.), each pixel’s “height” is estimated based on a local window. Depth precision requires fine, resolved textures on the object [43]. Increasing window size improves performance on low-feature objects, but increases lateral smoothing [44]. To counter this, wavelet methods [45] or adaptive window-size algorithms [46] have been suggested, but show object-dependent performance. Fine texture visibility is dependent on illumination direction, so the use of an LED ring is recommended.

The related depth-from-defocus technique estimates depth in a single image based on defocus. Its main advantage is removing the need for a focusing actuator, which comes at a performance cost compared to depth from focus. As Raman/LIBS spectrometers have a focusing actuator, it is not considered further.

The lateral resolution of depth from focus is limited by Equation (2) and lateral depth sampling further so by the algorithm choice. Blendowske [47] and Blayvas [48] have derived the depth resolution limit for a single-lens camera. Generalizing the expression in [47] for short working distances yields:

(10) $$\delta z=0.61\frac{NA_{out}}{NA_{in}^{2}}\sqrt{{\left(p_{x}\right)}^{2}+{\left(\frac{\lambda }{2NA_{out}}\right)}^{2}}$$

where *p_x_* is the pixel spacing. The achievable performance depends on noise, object heterogeneity and choice of algorithm [48]. The square-root term is assumed to be the “circle of confusion,” and the equation resembles the *DoF* Equation (4).

The measurement volume is limited laterally by Equation (2), while the depth is extendable to the range of the refocus. The scanning increment Δ*z* should approximately equal *δz*, but this depends on noise and algorithm choice. Since object-space telecentricity is prohibited, rectification algorithms must account for depth-dependent pixels shifting in object space. DFF requires the baseline imager and an LED measurement ring.

### 3.6. Confocal Microscopy

Scanning confocal microscopy involves 2D lateral plus 1D axial scanning of an illumination spot through the measurement volume, whilst the spot remains confocal to a pinhole within the instrument. The confocality with the pinhole passes on to detection of only light from a small region around the focus of the illuminated spot. For each lateral pixel, the intensity profile behind the pinhole follows a predictable response during axial scanning, with a peak when the surface is in focus. For each axial object coordinate, the confocality produces a depth section. Hahn [49] provides an overview of the variants in confocal microscopy for surface topography. Improved contrast and optical sectioning [50] have driven the technique’s widespread usage.

Confocal laser scanning microscopy (CLSM) uses a laser source for shorter integration time and insensitivity to ambient light. Multispectral depth maps can be made from spectrally separated channels and multiple light sources. Lateral scanning may be avoided by parallelization of lateral measurements. Care must be taken as this induces cross talk between neighboring pixels, with severity depending on the approach taken. Multiview methods [51] use pinhole arrays to simultaneously capture depth-sectioned images, but cannot be compactly integrated with a laser spectroscope (see Appendix E). Line-field scanning methods [52] use confocal slits in the illumination and detection paths to parallelize one scan axis, but at the cost of diminished contrast and resolution. Spectral encoding methods [53] can also parallelize measurements in one axis by dispersing a broadband illumination spot in one axis (lateral) and replacing the point detector behind the pinhole with a spectrometer. Spectrally encoded slit confocal microscopy (SESCoM [54]) combines line-field with spectral encoding to capture depth sections without lateral scanning.

Confocality is used in spectrometers to increase SNR, and thus CLSM has miniaturization potential with confocal spectrometers. If spectral encoding occurs over the wavelength range of the host spectrometer, the entire spectrometer subsystem (slit, grating, objectives, detector array and electronics) can be shared. Unfortunately, the dispersion requirements for detection optics for spectroscopy and SESCoM are contradictory.

**Figure 4:**
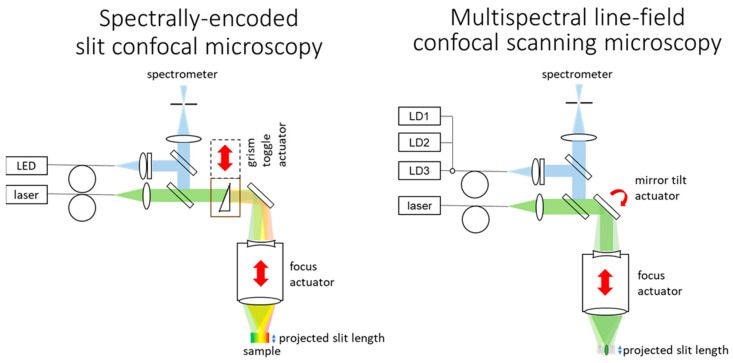
Spectrally encoded slit confocal microscopy (SESCoM) and multispectral line-field confocal scanning microscopy configurations for inline laser spectroscopy. LD: laser diode.

An active system that can toggle on/off on-axis dispersion allows switching between SESCoM and LIBS/Raman spectroscopy. This allows topography measurement with the spectrometer itself, as shown in Figure 4. Disadvantages of this approach include difficulty with colorful objects, the mass, power and risk of the toggle actuator, and preclusion of a multispectral measurement. A trade-off with a multi-laser, multispectral-capable line-scanning approach is required.

In either case, the number of frame captures is very large, as dictated by measurement volume, the depth and lateral sampling. As with depth from focus, the lacking object-sided telecentricity must be corrected in software.

The achieved axial resolution depends on the optical transfer function, the scanning precision and step size, noise, and algorithms. Fitting a curve to the axial intensity improves axial resolution such that it is limited by measurement and experimental noise rather than the diffraction limit. For this reason, experimentally measured uncertainty is often specified as a substitute for resolution. Repeatability or axial resolution can be much smaller than that predicted by FWHM of the optical transfer function [55], e.g., Jordan [56] reported 20×–50× improvement over the axial FWHM for mirror surfaces. We estimate the depth resolution as the theoretical FWHM divided by a factor *k* of 12, which depends on measurement noise and the topography. The achievable depth resolution is thus estimated as [57]:

(11) $$\delta z\approx \frac{2}{k\sqrt{2}}\frac{\;\lambda }{NA_{in}^{2}}\;$$

A larger pinhole diameter broadens the observation transfer function, trading resolving power for signal contrast [50]. The spectrometer autofocus scanning mechanism is exploited for CLSM and its mechanical uncertainty translates to depth uncertainty. The expected lateral resolution along the slit is given by Equation (2) and is improved by $\sqrt{2}$ across the slit [50]. The lateral measurement extent can be equal to that of the baseline imager. The axial scan range of the spectrometer limits the measurable object height. The depth scan increment Δ*z* should allow several samplings of the axial response function FWHM for robust fitting of the axial response function to noisy measurements. We use six samplings and *k* = 12 in this work, so Δ*z* should be twice the planned depth resolution (Equation (6)). If the spectrometer is used for topography measurement, the FOV requirements drive an increase in the diameter of the spectrometer optics, increasing their mass.

SESCoM requires a grism, toggle mechanism, LED with cylindrical lens and an amplitude-division beam splitter. A multispectral confocal line-scanning microscopy configuration is similar, with a mirror and tilt-scan mechanism replacing the grism and toggle mechanism and multiple laser diodes replacing the LED. Fluorescence, if present, allows use of the spectrometer excitation laser, avoiding the laser diodes and beam splitter and associated transmission losses and speckle noise.

### 3.7. Coherence Scanning Interferometry

Coherence scanning interferometry, reviewed in [58], uses localization of interference across optical path length difference (ΔOPL) to determine the topography or tomography. The approach is well known in biomedical applications as optical coherence tomography (OCT). Inline, partially coherent illumination leads to detectable interference only about the position where the ΔOPL between reference and object arms is below the coherence length of the light. For rough surfaces, the location of the contrast peak of the interference envelope infers surface height from the measurements. The setup for full-field measurement typically involves a Michelson, Mirau or Linnik interferometer with broadband Köhler illumination [58]. The noteworthy advantage of coherence scanning techniques is that the depth uncertainty is decoupled from imaging NA, although the robustness of sloped surface measurement increases with object-space NA and dispersion correction [59], limiting miniaturization potential.

**Figure 5:**
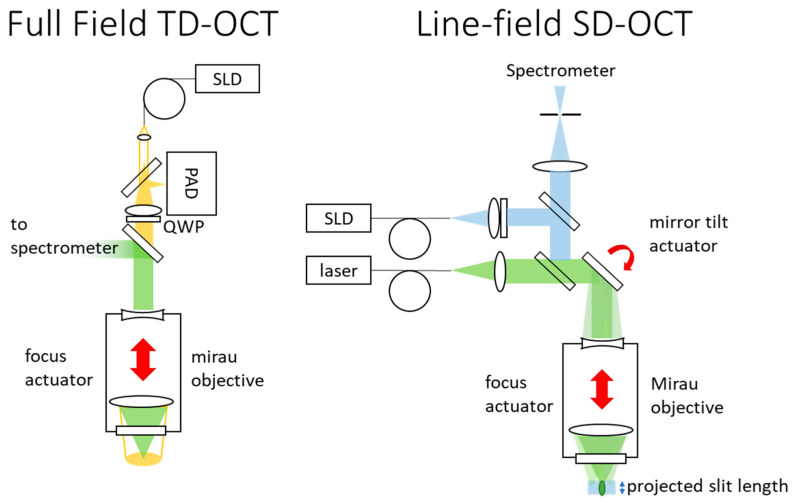
Schematics of full-field TD-OCT and line-field SD-OCT. SLD: super-luminescent diode; PAD: polarization array detector; QWP: quarter-wave plate.

Spectral domain OCT (SD-OCT), shown in Figure 5, combines backscattered broadband light with a reference signal to measure spectrally encoded depth with the spectrometer. The ΔOPL is scanned spectrally, yielding superior sensitivity. A line-field setup may make use of the existing slit spectrometer, which provides the sensitivity gains of confocal detection [60]. However, scanning is needed in one lateral axis and sensitivity decreases with increasing object depth [61]. The shared usage of the spectrometer dictates using the Raman/LIBS spectral bandwidth for topography measurement, bringing two constraints. Firstly, spectroscope transmission is lost due to beam splitting. Second, the depth measurement range of a line capture is limited to a fraction of a millimeter. Fusion of captures from different depths [62] and 1D lateral scanning are needed for sufficient measurement volume. The single-shot height measurement for each pixel improves robustness and simplifies telecentricity corrections.

Swept-source OCT uses a spectrally swept narrow-bandwidth illumination and an interferometric imaging detector to allow full-field mechanism-free 3D imaging within the DoF [63]. Spectrally swept sources based on VCSEL and fiber lasers have space heritage [64]. Coherent speckles, chromatic aberrations and pixel cross talk occur as areas of the image become defocused, restricting the measurable depth range [60]. SS-OCT will be worth further consideration when space-qualified MEMS-based tip/tilt 2D scanners become commercially available, allowing highly miniaturized, fiber-based OCT systems.

Mechanical scanning allows the focal plane and the zero-ΔOPL plane to remain aligned during ΔOPL scanning, removing a defocus limitation to full-field measurement, though defocus within the FOV still causes cross-talk errors. This technique has several names, including time-domain OCT (TD-OCT), white-light interferometry, and coherence scanning interferometry. Instrument mass and interface limitations preclude translating the entire instrument or object, while scanning the reference mirror alone is insufficient. Using a Mirau or Michelson objective works, but reduces working distance and increases mass, particularly if they must have variable focus.

To characterize the coherence envelope and localize its peak, a few measurements per half-period of the fringe pattern are needed. Measurement parallelization with polarization-multiplexed phase shifting [65], multiple reference arm reflection planes [66] or off-axis holographic setups [67] are possible. Increasing object depth within the FOV leads to detrimental speckles and defocus-induced cross talk. Reducing coherence length helps this, but proportionately lengthens measurement time. The larger the working distance and longer the integration time, the more likely instabilities will destroy interference visibility. As many z-plane coherence measurements are used to evaluate the height of a single pixel, software telecentricity correction is complicated.

Measurements are highly redundant, as most pixels in a plane of measurement contain no information. The number of captures required for polarization-multiplexed TD-OCT is the measurement range divided by the sampling period, which must be sufficient to characterize the coherence envelope. Multispectral depth maps are extractable from spectrally scanned datasets [68] or by merging in-focus images captured with sequential LED illumination.

Lateral resolution is given by Equation (2), while axial resolution can be given as the coherence length for both spectrally and mechanically scanned approaches, which is given for a light with a Gaussian spectral distribution as per [69]:

(12) $$\delta z_{FWHM}=\frac{2ln2}{k\pi }\frac{\lambda_{c}^{2}}{\Delta \lambda }$$

where *k* is a factor of improvement in the resolution achieved by fitting a theoretically known curve to the measurements. As per the discussion in Section 3.2, the local roughness may cause uncertainty higher than this value. For fitting to noisy data, the TD-OCT depth scan increment Δ*z* should be approximately twice the axial resolution. The measurement volume is laterally limited by the imager FOV (Equation (5)). The depth measurement range is the autofocus range. Depth scan increments for *SD-OCT* are limited by defocus and spectrometer resolution [69]:

(13) $$\Delta z_{SD-OCT}=\min\left[DoF,\frac{1}{4}\frac{\lambda_{c}^{2}}{\Delta \lambda }Ν\right]$$

where *λ_c_* is the central wavelength, Δ*λ* is the wavelength range and *N* is the number of sample points across the spectral width. A TD-OCT setup requires the baseline imaging components, an inline SLD, beam splitter and collimation objective, a Michelson/Mirau objective. Line-field *SD-OCT* requires an additional cylindrical lens and a mirror tilt-scan mechanism. A quarter waveplate and micro-polarizer array on the detector are needed for polarization multiplexed for TD-OCT.

### 3.8. Multiwavelength Digital Holography

Digital holography (DH) (see [70] for an overview), captures interferograms (“holograms”) of object and reference fields at the detector array of an interferometer. Following digital post-processing, an array of complex numbers representing the complex object field is extracted from real valued interferograms. From here, the amplitude and phase can be calculated for any point in space, unlocking large measurement volumes from a single shot. Numerical processing may further include refocusing, aberration correction, filtering and noise reduction [70]. Whilst not strictly needed, lenses are useful for adapting the object field to match the limiting spatial bandwidth of holographic detection.

Phase-shifting DH extracts the complex object field from temporally separated holograms with known [71] or unknown [72] phase shifts of a reference field. Single-shot approaches such as off-axis DH or polarization-multiplexed phase-shifting DH [73] are sensitive to vibration, but suffer a loss of spatial bandwidth.

Smooth objects allow direct evaluation of the height from the phase calculated at the “object plane”. This requires a 2π-moduli unwrapping algorithm, which have been reviewed [74,75,76]. Rough surfaces yield “random” phase at the object plane, causing speckle noise in the reconstructed object plane. Whilst still allowing for depth-from-focus evaluation [77] to an extent, the precision of direct phase evaluation approaches is much better, so techniques were developed to reduce speckle by various means, reviewed in [78].

Multiwavelength DH (MWDH) [79] allows precision topographic measurement despite speckled holograms. MWDH works by varying the illumination wavelength between sequential hologram recordings. The subtraction of object-phase maps between sequential captures removes the random speckle phase, producing a wrapped phase map corresponding to a difference in wavenumbers $\Delta k=k_{1}-k_{2}=2\pi \left(\frac{1}{\lambda_{1}}-\frac{1}{\lambda_{2}}\right)=2\pi /\Lambda$. The synthetic wavelength *Λ* can be much larger than a constituent wavelength:

(14) $$\Lambda =\frac{\lambda_{1}\lambda_{2}}{\left|\lambda_{1}-\lambda_{2}\right|}$$

The unambiguous height range as well as phase errors are multiplied by $\Lambda /\lambda_{1}$. Errors are reducible to original levels if the phase difference map guides the unwrapping of an original, unless the multiplied phase error exceeds 2*π*. Using a multitude of wavelengths, cascading processing of phase maps can extend the unambiguous depth range, increase precision and reduce the sensitivity to phase error [79].

Varying wavelength between captures induces speckle decorrelation, adding phase errors in the phase difference map. Decorrelation increases with object slope, roughness and limited aperture [80]. As an estimate for flat surfaces, the *Λ* should be more than 20× the areal RMS roughness. This limits the minimum *Λ* and subsequently the depth resolution. Measurements at different wavelengths may be parallelized with angular multiplexing [81] at the cost of spatial resolution. Translations between measurements (i.e., drift) can be compensated [82,83]. To reduce decorrelation errors, instrument stiffness and measurement speed should be increased.

**Figure 6:**
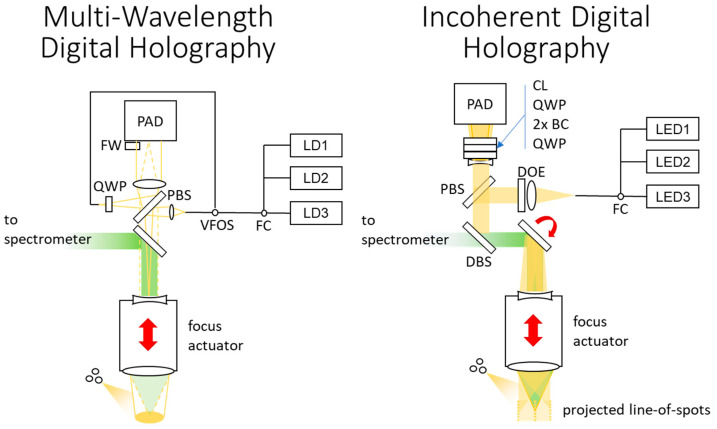
Schematics of polarization multiplexed digital holography and incoherent digital holography based on linear conoscopy. CCD: charge-coupled device, PAD: polarization-array detector, FW: Fizeau wedge, VFOS: variable fiberoptic switch, FC: fiber combiner, LD: laser diode, LED: light-emitting diode, CL: cylindrical lens, QWP: quarter-wave plate, BC: birefringent crystal, BS: beam splitter, DBS: dichroic beam splitter, DOE: diffractive optical element.

The wavelength differences Δ*λ* should be known to the picometer [84], suggesting in situ wavelength characterization. An inline temperature-controlled wedge plate in the reference beam path may work, as shown in Figure 6. Spatially coherent, narrow bandwidth sources with power and frequency stability are required. Single compact tunable sources such as single-lateral-mode laser diodes [85] or tunable fiber lasers are possible.

An algorithm for minimizing the number of measurement wavelengths needed for a given object is given in [86]. As an example, a 2 mm-depth range could be measured with 800.00 nm, 800.14 nm, 800.90 nm and 839.99 nm if the measurement phase error is 1/30.

Volume-scattering samples are generally difficult to measure optically, but as a coherent technique, they are especially challenging for MWDH. This implies MWDH is not suitable for icy samples. To achieve multispectral topography, MWDH setups can be simply used as imagers with additional LED illumination.

An advantage of MWDH is good spatial resolution across a large depth range, from few measurements without moving parts. Lateral resolution is limited by the coherent light version of Equation (2). Polarization-multiplexed phase shifting doubles the pixel spacing. Non-imaging setups are constrained by the minimum fringe spacing that must be adequately sampled, as well as by the effective numerical aperture of the hologram converted into object space. To estimate the depth resolution, the phase evaluation of the smallest synthetic wavelength leads to the approximation *δz* = *Λ_min_*/100.

The lateral measurement range is that of a MWDH imaging system and limited by Equation (5). The unambiguous measurement depth range Δ*z* is limited to *Λ_max_*/2. Defocus does not affect measurement range, but longer numerical propagation to obtain focus leads to more approximation and characterization errors.

Polarization-multiplexed multiwavelength DH in an imaging configuration requires the baseline imager components, a micro-polarizer array for the detector, two or three thermally controlled laser diodes, optical switches (e.g., fiber-based 3 × 1 combiner and a fiber-based variable switch), a quarter waveplate, two lenses, a Fizeau plate and a polarizing beam splitter.

### 3.9. Incoherent Digital Holography

Incoherent digital holography (IDH, see [87] for overview) involves minimizing the optical path length differences to allow use of low-coherence light and prevent speckle. Self-interference of the object field duplicates achieves this, akin to shearing interferometry. Spatial light modulators may apply phase-shifted field-curvature differences between the object field copies before detection [88]. Such common-path self-referencing interferometers are robust and even implementable with passive optics arranged as an axial-shearing interferometer [89,90], geometric phase optics [88] or, under the name “conoscopy”, with birefringent crystals [89].

The curvature difference produces interferograms resembling a Gabor zone lens for each object point, whose phase is inversely proportional to the square of the object distance in detector space [89,90]. The quadratic dependence leads to non-linear mapping from hologram to object space and non-linear object space resolution. This is avoided with a lateral shearing arrangement [91], also known as linear conoscopy [92], that produces linear phase variation across the detector in the form of Δ*Φ* = 2*πK*_lin_*x*/*z_H_* for an illuminated line on the object. The period *T* of the resulting 1D sinusoidal interferograms is proportional to the height of the object point on the line. Varying the parameters of crystal length, angle and material affects *K_lin_*, scaling depth resolution and range. Another issue is dynamic range of detection for full-field self-referencing IDH [87]. Overlapping interferograms from nearby object points are incoherently added at the detector, causing low visibility of individual interferograms and a bias towards bright object points [93], burdening the limited detection dynamic range. Structured illumination can reduce the number of contributing object points, while cylindrical lenses can prevent their overlap at the detector. For this reason, scanned point- or line-field illumination can be more practical than full-field IDH measurement.

A linear conoscopy setup with line-field illumination is shown in Figure 6. Utilizing an inline projector may avoid scanning mechanisms, but it decreases lateral resolution. The low coherence requirements enable projection pattern illumination with narrowband LEDs. Multispectral topographies could be obtained by merging multiple results from different illumination sources. The number of required measurements for a linear conoscopy setup is the number of captures across the lateral scan axis.

**Figure A1:**
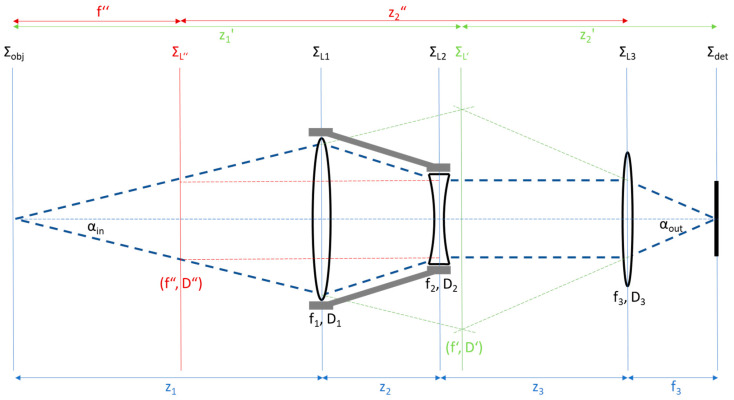
Infinite-conjugate-objective imaging system, modeled as a three-thin-lens system, as well as beam paths for equivalent one-lens (L’) and two-lens (L”) models.

The lateral resolution along and across the line in object space differs. Across the line, the lateral resolution is limited by the illumination spot:

(15) $${\delta x}_{across}=\frac{0.61\lambda }{NA_{ill}}$$

where *NA_ill_* is the illumination numerical aperture, which may be reduced by design to better support deep objects. Along the line, the resolution conforms to Equation (2). The depth resolution is limited by the uncertainty *δT* of the period of the fitted sinusoidal function to the pixelated interferogram with phase Δ*Φ =* 2*πK*_lin_*x*/*z_H_*. The resolution limit in object space can be estimated as:

(16) $$\delta z=\frac{{Κ}_{lin}\delta T}{M_{IDH}2}$$

where *M_IDH_*^2^ is the axial magnification when a weak negative lens replaces lens 3 in Figure A1. The uncertainty in the factors constituting *K_lin_* (i.e., temperature dependence of refractive indices) can be relevant. Low-coherence illumination reduces the fundamental speckle limitations otherwise applicable to lateral shearing interferometry [94]. The lateral measurement extent is scanning range- or FOV-limited (Equation (5)). The depth measurement range Δ*z* is limited by the depth of field of the illumination optics.

Polarization-multiplexed line-scanning linear conoscopy requires shearing optics (e.g., birefringent crystals), two quarter-wave plates, a cylindrical lens and a negative lens, and a detector array with micro-polarizer array. For illumination, it needs a polarizing beam splitter, a DOE and narrowband LEDs/LDs. Finally, a 1D scanner is needed to scan the full measurement range.

With both requirements and candidate 3D technologies well understood, we now present performance, mass and power estimations and discuss technology selection.

## 4. Comparison of the 3D Technologies

The best choice of technology is extremely dependent on the requirements of the specific application. In this section, the comparison is discussed from three different perspectives: spatial performance of topography measurement, SWaP, and robustness.

### 4.1. Spatial Performance of Topography Measurement

It is strategically interesting to consider if one technology could be used in all rover configurations. To check for this, we eliminate incompatible techniques for each rover configuration. To this end, Table 2 presents the estimated optical performance of each technique in each of the rover mounting configurations, using the design parameters given in Appendix C and the equations presented in previous chapters.

For fair comparison, all techniques’ performance calculations were estimated with identical parameters, as far as this was possible. Therefore, optimizations for each technique may still be possible. The equations for all techniques are provided to enable the reader to compare techniques based on their specific needs.

Table 2 shows that an aberration-free baseline imager is able to fulfill the lateral 2D imaging requirements given the design parameters used in this study, though the DOF cannot capture sharp images of whole objects in a single image.

For forward-looking instruments, inline PS-FPP would meet requirements if phase uncertainty is as low as 1/100. Coherence scanning techniques (TD-OCT and SD-OCT) at long range would require the complexity and mass of variable-focus Mirau objectives as well as high-power illumination to reduce integration times and thus sensitivity to motion. Like TD-OCT, confocal techniques are challenging at range as they require high precision in the variable focal length objective. Off-axis stereography and fringe projection techniques with long working distances prohibit compact integrability with an external scanner and require additional refocusing mechanisms. For the long distances of the front-looking configuration, MWDH and IDH excel.

For downward-looking and near-contact instruments, a translating fixed-focus objective becomes plausible, allowing coherence and confocal scanning techniques. The split aperture of inline FPP prevents the fulfillment of lateral sampling requirements. Depth from focus may meet depth-resolution requirements under ideal conditions with a different magnification, but the smoothed (sparse) depth sampling and real objects and aberrations are unresolved problems.

These considerations reveal MWDH and IDH to be techniques most suited to scaling to different sizes. MWDH relaxes aberration requirements, reducing complexity of larger, longer working distance objectives.

### 4.2. Mass and Power Estimation

The mass or power consumption for each of the required components was summed for each candidate technology. References were taken from the heritage RAX instrument, which is comparable only in size to the “bottom-mounted panel” use case. The estimations, shown in Table 3, are qualitative, since detailed designs of optical, structural, control electronics and power systems are needed for quantitative estimation, but outside the scope of this work.

The symbols “○”, “◑”, “●”, “●○” to refer to estimations of 0–0.5×, 0.5–1×, 1–1.5×, and 1.5–2× more mass than the mass estimate for the baseline imager, or estimated power consumed when compared to the RAX Raman measurement mode mean power consumption. The symbol “√” and “×” mean requirements (“Inline” for inline measurement or “ST” for maintained spectrometer transmission) are met and not met respectively. The restricted mass increase requirement will be challenging to meet with mass ratings of “●” and “●○”.

The heaviest techniques are the off-axis stereography, FPP and IDH, which require additional actuation and detection/projection. Inline FPP saves mass using the spectrometer autofocus in projection. A similar mass is achieved by TD- and SD-OCT, which are inline but need heavier Mirau objectives. MWDH uses a large number of components, so the end-result is similar. Finally, using the spectrometer itself to measure topography demands across-slit scanning, resulting in negligible mass savings compared to using a baseline imager.

There are no power savings in utilizing the host spectrometer for topography measurement instead of the baseline imager if spectra are also recorded on a 2D detector array. This is done to allow slit imaging to support autofocusing and spectroscopic measurements from all points along the slit. Since line-scanning control and actuation add power, topography measurement with baseline imagers has lower power consumption than those with line scanning. Techniques with temperature stabilization can expect the highest power consumption, with MWDH requiring individual thermal control elements for each laser source resulting in the largest power consumption. Power is indicative only of a possible peak power draw, while total energy consumption requires estimation of total measurement time. It may well be that MWDH has the highest power draw but fastest measurement and lowest total energy consumption. To estimate energy requirements requires estimates of integration times, which is related to the illumination (laser/LED, full-field/scanned), but is beyond the scope of this work.

Instrument size estimation is strongly affected by detailed design and not presented. Techniques utilizing the host spectrometer may be smallest, while baseline imager techniques would be smaller than interferometric techniques. Off-axis techniques have larger but spatially separated volumes.

Overall, depth-from-focus and plenoptic technologies, and to a lesser extent confocal approaches, perform well on SWaP, while off-axis stereography and FPP approaches perform worst.

### 4.3. Measurement Robustness

Optical measurement of topography of rocks and minerals can be particularly challenging. Adhered, unresolvable regolith can aid topography measurements of otherwise specular surfaces by increasing surface scattering. The increased apparent roughness is however detrimental to the depth uncertainty of interferometric-based techniques. Transparent, sloped surfaces, and volume and multiple scattering further complicate optical measurement. Blocking photons before detection makes confocal techniques uniquely robust. Volume scattering increases the apparent height distribution of scatterers at each pixel. Coherence scanning techniques have a depth-discriminating “gate” like confocal techniques, but scatters within the coherence length still contribute noise. The incoherent illumination in IDH makes it more robust than MWDH, which suffers roughness and slope-related speckle decorrelation errors.

Specular, sloped surface issues are lessened for all optical techniques by increasing collection NA, so near-contact instruments should be most robust. Strong surface slopes are an issue for defocus-induced pixel cross talk, which affects confocal and coherence scanning techniques, whose long-distance applications are less robust. Simulation is required to assess cross-talk impact for a given optical design.

Depth from focus is not universally applicable due to specific texture size requirements. It should be considered as a bonus technique available to any configuration utilizing the baseline imager. For example, while triangulation techniques suffer from shadowing, depth from focus could be applied via a software change to augment occlusions.

Motion is ruinous to interference-based techniques, even for single-shot approaches. Increasing source-illumination power helps. TD-OCT is impractical if the rover causes or is subject to any vibration during measurement.

In following these considerations, confocal approaches (if cross talk is low), FPP and IDH appear to be the more generally robust approaches for the expected sample types.

## 5. Conclusions and Outlook

For inline topography measurement with rover-borne laser spectrometers, the best technique is strongly dependent on rover mounting location and prior knowledge of the samples to be measured. Inline FPP and MWDH are strong candidates for future investigation as techniques suitable for the application that do not require additional scanning mechanisms and can both operate in depth-from-focus mode or multispectral imaging mode with changes only to software. An inline PS-FPP design must be proven to accommodate split-aperture aberrations with a long-working-distance objective while precisely determining fringe phase on distant, dark objects. MWDH requires the development of an inline wavemeter and a proven miniaturized optical design. IDH is another good candidate, where combinations of partially coherent illumination and LCD displays may yield mechanism-free, highly robust measurement. If a 1D tilt scanning mechanism is available and the host spectrometer can use a line-array detector, multispectral line-field scanning microscopy becomes attractive. The next steps would be to determine the limits imposed by cross talk. This would help spectrally encoded slit confocal microscopy too, which is low mass. If the working distance can be small, more standard approaches like coherence scanning interferometry may be most suitable.

## Figures and Tables

**Table 1:** Summary of the spatial requirements for each mounting configuration.

Mounting Configuration	Max. Working Distance (mm)	Working Distance Range * (mm)	Resolution ** (µm)	Minimum Measurement Volume (mm^3^)
Front/side panel	500	150	30	10 × 10 × 10
Bottom panel	150	30	10	2 × 2 × 2
Contact window	10	3	5	1 × 1 × 1

* The working distance range is also the range of the spectrometer autofocus. ** Lateral as well as axial direction.

**Table 2:** Measurement performance estimates of optical topography techniques in three rover-relevant configurations. δx and δz are achievable and require spatial sampling, Δx and Δz* as lateral and depth measurement range. Red text highlights unmet requirements. * Depth range measurable without fusing results.

		Front Panel-Mounted NAin = 0.125, WD = 500 mm				Bottom Panel-Mounted NAin = 0.125, WD = 150 mm				Contact Window NAin = 0.2, WD = 10 mm			
	Requirements	δx (µm)	δz (µm)	Δx (mm)	Δz* (mm)	δx (µm)	δz (µm)	Δx (mm)	Δz* (mm)	δx (µm)	δz (µm)	Δx (mm)	Δz* (mm)
Technology		(<30)	(<30)	(>10)	(>10)	(<10)	(<10)	(>2)	(>2)	(<5)	(<5)	(>1)	(>1)
Baseline Imager		3.42	-	26.4	0.24	3.42	-	26.4	0.08	2.14	-	11	0.025
Depth from Focus		3.42	18	26.4	0.02	3.42	18	26.4	0.018	2.14	6.15	11	0.006
Multi-λ Confocal Line Scan		3.42	4.62	26.4	0.009	3.42	4.62	26.4	0.009	2.14	1.82	11	0.004
Spectral-Encoded Confocal Slit		3.42	4.62	26.4	0.009	3.42	4.62	26.4	0.009	2.14	1.82	11	0.004
Stereography (Off-Axis)		3.42	23.6	26.4	0.24	3.42	11.4	26.4	0.08	2.14	7.12	11	0.025
PS-FPP (Off-Axis)		3.42	3.46	26.4	0.24	3.42	1.74	26.4	0.08	2.14	1.74	11	0.025
PS-FPP (Inline)		6.83	16	26.4	0.24	6.83	16	26.4	0.08	4.27	10	11	0.025
Full-field TD-OCT		3.42	2.07	26.4	0.004	3.42	2.07	26.4	0.004	2.14	2.07	11	0.004
Line-scan SD-OCT		3.42	0.1	26.4	0.24	3.42	0.1	26.4	0.08	2.14	0.1	11	0.025
Multi-λ DH		5.25	3.91	26.4	12.5	5.25	0.17	26.4	2.31	3.28	0.39	11	1.25
Incoherent DH (Linear Conoscopy)		21.35	2.57	26.4	1.50	8.54	2.57	26.4	0.20	4.27	1	11	0.050

**Table 3:** General performance comparison of optical topography technologies. * All optical techniques are challenged by low backscattering surfaces.

Technology	Mass	Power	No. CCD Captures	Inline	ST	Sample Difficulties	Other Issues
Depth from Focus	○	○	100–500	√	√	Low textures	Lateral smoothing
Multi-λ Confocal Line-scan	○	●	200,000–1,000,000	√	×	Defocus cross talk	Autofocus precision 1D scanner No zoom support
Spectral-Encoded Confocal Slit	○	◑	200–1000	√	×	Colorful objects Defocus cross talk	Autofocus precision Not multispectral No zoom support
Stereography (Off-Axis)	●○	◑	10–40	×	√	Low textures	Refocusable 2nd camera
PS-FPP (Off-Axis)	●	●	10–40	×	√	Low backscattering surfaces *	Refocusable projector SLM/display usage
PS-FPP (Inline)	◑	◑	10–40	√	√	Low backscattering surfaces *	Split-aperture aberrations SLM/display usage
Plenoptic Camera	○	○	25–40	√	√	Low textures	High depth uncertainty
Full-Field TD-OCT	◑	●	2000–4000	√	√	Defocus cross talk Volume scatter	No variable-focus Mirau Vibration sensitivity Autofocus precision
Line-scan SD-OCT	◑	●	10,000–20,000	√	×	Defocus cross-talk volume scatter	No variable-focus Mirau 1D scanner Vibration sensitivity No zoom support
Multi-λ DH	◑	●○	4–10	√	√	Volume scatter	Inline λ-meter needed Speckle decorrelation
Incoherent DH (Linear Conoscopy)	●	○	5000–20,000	√	√	Volume scatter	Unconventional 1D scanner Low SNR

**Figure A2:**
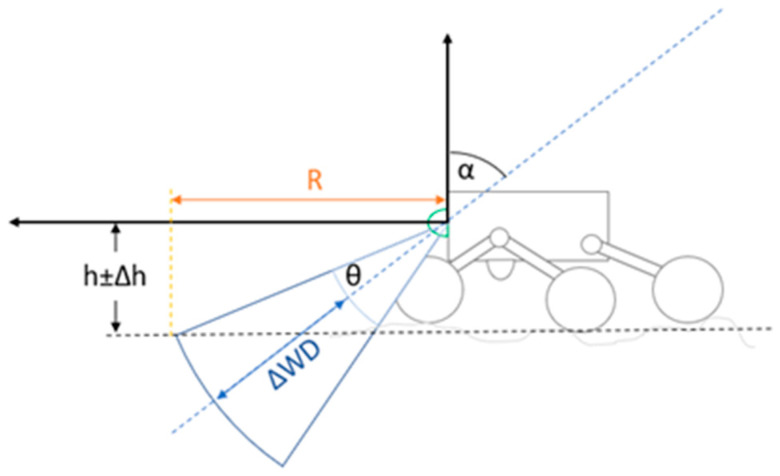
Definition of rover layout variables for front-, side- and downward-facing instruments.

**Figure A3:**
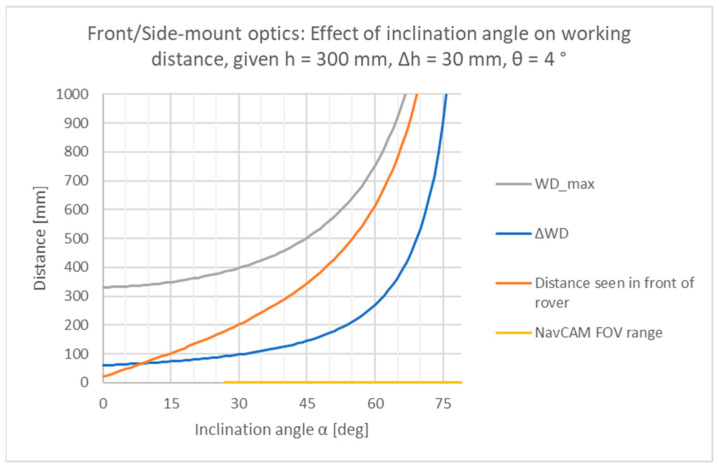
Calculated working distance parameters for front- and side-mounted instruments.

**Figure A4:**
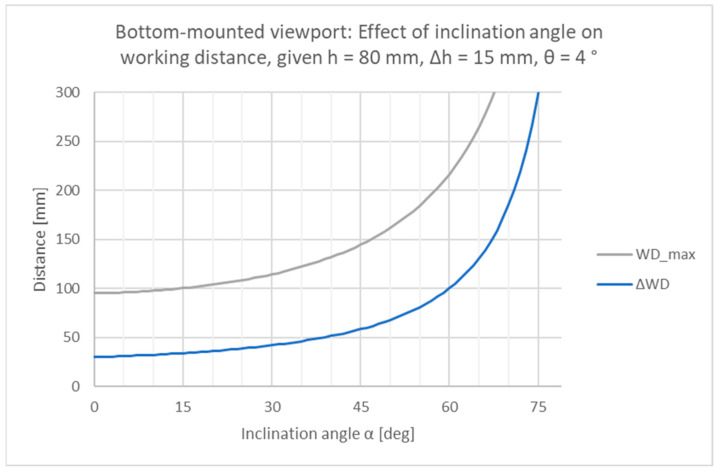
Calculated working distance parameters for bottom-panel-mounted instruments.

**Figure A5:**
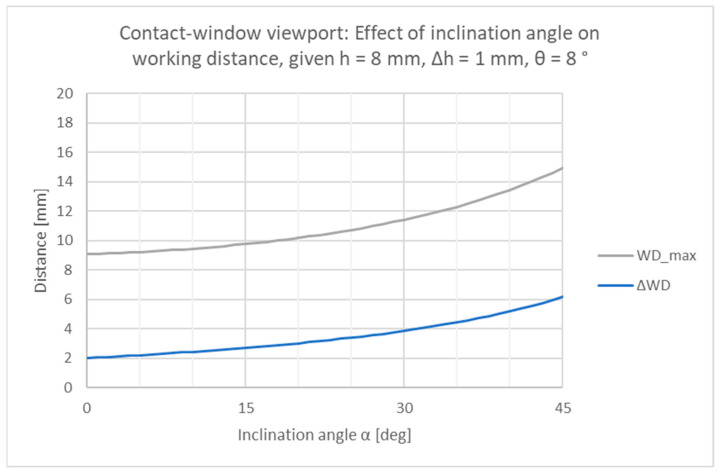
Calculated working distance parameters for in- or near-contact measuring instruments.

## Data Availability

Data are contained within the article.

## Appendix A. Optical Model of a Baseline Imager

Figure A1 shows the optical imager model used in this work. The thick dashed line is the optical path of the three-lens model, which is the highest-fidelity model in this study. Light from a point in the object plane Σ_obj_ traverses the working distance z_1_ until refracted by the positive lens at Σ_L1_, the first of the focusing objectives. A negative second lens at Σ_L2_ outputs a collimated beam from the focusing objective. A positive lens at Σ_L3_ with its focal plane at the detector plane Σ_det_ provides a focused image. The three-lens model is complex enough to estimate the size and weight of components with some accuracy, but cannot be directly applied to performance-estimating equations in the literature. For this purpose, two additional models are created: a two-lens system model replaces L_1_ and L_2_ with L” at a distance equal the focal length f” from Σ_obj_, and a one-lens system model replacing all the lenses with L’ at distance z_1′_ from Σ_obj_. The equations to calculate equivalent one- and two-lens system parameters from a three-lens system are given in Appendix D.

Some techniques benefit from object-side telecentricity, which is realized by placing an aperture in the back focal plane of the object-facing objective. However, the same objective must accommodate the spectrometer. In order to preserve the sensitivity of the spectrometer, the collection NA should not be compromised, so the spectroscopic light must be separated before the exit pupil. Further, a variable-focal-length objective requires a moving aperture. For these two reasons, object-side telecentricity is not considered in this work.

## Appendix B. Working Distance, Measurement Volume Dependence on Rover Mounting Configuration

Figure A2 shows the relationship between working distance variation ΔWD, instrument height above the object h and its variation Δh, inclination α and scannable range θ.

The range in working distances to be covered by the instrument, assuming a circular FOV, can be given as the difference between minimum and maximum working distances, varying over the FOV:

(A1) $$\Delta WD=WD_{max}-WD_{min}=\frac{h+\Delta h}{\cos\left(\alpha +\theta \right)}-\frac{\left(h-\Delta h\right)}{cos(\alpha -\theta )}$$

(A2) $$\Delta WD=\frac{4\left(hsin\left(\alpha \right)\sin\left(\theta \right)+\Delta hcos\left(\alpha \right)cos\left(\theta \right)\right)}{\cos\left(2\alpha \right)+\cos\left(2\theta \right)}$$

where *h* is the nominal height of the instrument above the ground plane, Δ*h* is the expected variation in actual height, *α* is the inclination of the optical axis from vertical, and *θ* is the FOV half angle. The effect of *α* and *θ* on Δ*WD* is identical. The autofocus of the instrument must scan the Δ*WD* range. For a front- or side-panel-mounted instrument, mounted 300 mm above the ground, Figure A3 shows that an inclination angle *α* of 45 degrees is within the rover navigation camera’s FOV. Combined with a 4° FOV half angle, the maximum working distance needed would be 500 mm and the maximum distance seen in front of the rover is 345 mm.

A bottom-mounted viewing port for the instrument implies that the rover can drive over the sample, reducing the possible height variation Δ*h*. Applying the configuration of the RAX instrument in the MMX rover to the same equations yields Figure A4. A maximum working distance of 150 mm and working distance range of 60 mm would allow for ±45° scanning, while a working distance range of 30 mm would suffice for ±4° scanning (any point of interest in original FOV).

For window-contact measurements, the working distance range must only cover the local height variation. Some focus variation is necessary to avoid measurement failures when the instrument has a recess in the line of sight. A height variation of 1 mm is assumed here, as with the RLS [25]. A maximum working distance of 10 mm allows ±8° scanning of the entire FOV, so targets of spectroscopic interest anywhere in the FOV could be scanned.

For correlation with other onboard camera images (e.g., navigation) the rectified contextual (topography) image should be more than 20 × 20 pixels in the other cameras image. Taking the MMX rover navigation cameras [95] as an example, this corresponds to an angular FOV of >20 mrad or measured area of over 10 × 10 mm^2^. Applying the same logic to the MMX wheel camera as an analogue for the bottom-mounted case yields a required angular FOV of >13 mrad or >2 × 2 mm^2^. The location of WD10 measurement is known, so no correlation with other images is needed. As fine resolution and large FOV are always both demanded, lessons from previous missions [96] have shown the topographic technique should support variable optical zoom. For ultimate accuracy of derived morphological information, the lateral and transverse sampling periods should match. The sampling uncertainty shall be equal to or less than the sampling period.

## Appendix C. Design Parameters Used in Calculations

**Table A1:** Design parameters used in calculations.

Technology	Parameter	Contact Window	Bottom Panel Mounted	Front Panel Mounted	Units
Imager	z_1_	10	150	500	mm
	f_1_	10.000	49.367	180.766	mm
	D_1_	4	37.5	125	mm
	z_2_	6	52	200	mm
	f_2_	inf	−21.585	−83.126	mm
	d_2_	4	11	36.7	mm
	z_3_	8	40	50	mm
	f_3_	10	18.3	61.2	mm
	d_3_	4	11	36.7	mm
	NA_out_	0.2	0.3	0.3	-
	NA_in_	0.2	0.125	0.125	-
	|M|	1.00	0.42	0.42	-
	pixel size	0.001	0.001	0.001	mm
Spectrometer	λ_spectometer, mean	0.00061	0.00061	0.00061	mm
Confocal	K	12	12	12	-
	λ_inline	0.0007	0.0007	0.0007	µm
off-axis tri.	β	33	33	16.5	°
	b	145	90	6	mm
inline tri.	β	5.73	3.58	3.58	°
FPP	T	0.1	0.1	0.1	mm
	δφ/2π	0.01	0.01	0.01	-
TD-OCT	λ_c_	0.75	0.75	0.75	µm
	Δλ	0.01	0.01	0.01	µm
FD-OCT	λ_c_	0.6075	0.6075	0.6075	µm
	Δλ	0.145	0.145	0.145	µm
	N	390	390	390	-
MW-DH	λ_0_	0.8	0.8	0.8	
	λ_1_	0.80025608	0.800138691	0.800025601	µm
	λ_2_	0.80102531	0.80090235	0.800102413	µm
	λ_3_	0.81672656	0.83998467	0.801641762	µm
	Λ_1_	2500	4615.384615	25000	µm
	Λ_2_	625	710.0591716	6250	µm
	Λ_3_	39.0625	16.80613424	390.625	µm
IDH	K_lin_	−155	−155	−155	-
	NA_ill_	0.1	0.05	0.02	-
	|MIDH|	5.56	3.47	3.47	-

## Appendix D. Equations to Convert Working Distances and Diameters into One-, Two-, and Three-Thin-Lens Paraxial Systems

Using the variables presented in Figure 3 of the main text, the focal lengths for a three-lens system can be calculated as:

(A3) $$f_{1}=\frac{z_{1}z_{2}D_{1}}{z_{1}\left(D_{1}-D_{2}\right)+z_{2}D_{1}}$$

(A4) $$f_{2}=z_{2}(1-\frac{D_{1}}{D_{1}-D_{2}})$$

while the numerical aperture is calculated as $NA_{in}=\frac{D_{1}}{2z_{1}}$ and $NA_{out}=\frac{D_{3}}{2z_{3}}$. For a two-lens system, $D_{L}^{\prime \prime }=D_{3}=D_{2}$, and:

(A5) $$f_{L}^{\prime \prime }=\frac{D_{2}}{D_{1}}z_{1}$$

(A6) $$z_{2}^{\prime \prime }=\frac{D_{L}^{\prime }-D_{3}}{2}(\frac{1}{NA_{in}}+\frac{1}{NA_{out}})$$

Finally, for a single-lens system:

(A7) $$f_{L}^{\prime }=\frac{\left(z_{1}+z_{2}+z_{3}+f_{3}\right)}{\left(1+\frac{NA_{out}}{NA_{in}}\right)\left(1+\frac{NA_{in}}{NA_{out}}\right)}$$

(A8) $$z_{2}^{\prime }=\frac{\left(z_{1}+z_{2}+z_{3}+f_{3}\right)}{1+\frac{NA_{out}}{NA_{in}}}$$

(A9) $$z_{1}^{\prime }=z_{2}^{\prime }(\frac{NA_{out}}{NA_{int}})$$

(A10) $$D_{L}^{\prime }=2NA_{out}z_{2}^{\prime }$$

## Appendix E. 3D Optical Techniques Precluded from This Study

### Appendix E.1. Hyperspectral Holography, or 3D Imaging Spectroscopy

From a polychromatic object field, extraction of fields of specific wavelengths is possible with wavelength-specific phase shifts [97]. This is generalized for broadband light by using a Fourier transform in a self-referencing setup, allowing spectral properties of the complex object field to be measured [98,99,100]. In such a way, a single FT spectroscope can measure topography and spectrometry.

Unfortunately, 3D spectroscopic measurement remains impractical. Memory constraints force a sacrifice in spectral or spatial performance. For topographic measurement, high spatial resolution is needed for holographic depth from focus, forcing a sacrifice in spectral resolution. However, poor spectral resolution combined with broadband scattering/emission from the object is ruinous to hologram quality. Superior SNR is achieved with multiple discrete illumination wavelengths (laser diodes) using wavelength-dependent phase shifts.

For spectroscopic measurement, spatial binning to tens of pixels per axis is required for manageable raw data amounts per scan. The loss in spatial resolution impedes identification and specificity and prevents holography, demanding in-focus 2D imaging. Raman and LIBS spectroscopy must also be in-focus with the surface, limiting their full-field applications to artificially flat (or axially scanned) objects.

The optimum is separate measurements of topography with a few wavelengths of interest from the entire FOV, then applying correspondence to a few discrete locations where the entire spectrum has been measured. Readout rates (camera- and interface-limited) will make it challenging to measure both multispectral topography and multilocation spectra within time and energy budgets.

### Appendix E.2. Plenoptic Camera

As stated in Section 3.3, stereography realized with a single camera by aperture division results in a focused [101] or unfocused [102] plenoptic camera.

An unfocused plenoptic camera has the MLA in the focal plane of the preceding optical system, with the detector in the Fourier plane of the micro-lenses. Unfocused plenoptic cameras achieve better angular or depth resolution than focused plenoptic cameras, obtained at a cost of lateral resolution. The projected lenslet pitch at the object is the lateral resolution at the object [103]. To resolve silt/sand in this application, the lenslet diameter D_4_ would have to be less than 30 µm, which raises aberration, manufacturing and detector pixel-size issues. For these reasons, unfocused plenoptic cameras are not further investigated.

In a focused plenoptic camera, each lenslet images the imaging plane of the baseline imager onto the detector, which creates micro-images of the scene from different perspectives. Object depth is derived from triangulation using the disparity of homologous features. Fine textures are required and the depth resolution and uncertainty are limited by the maximum angle of triangulation, which is a fraction of the numerical aperture of the autofocusing objective. This indicates that focused plenoptic cameras are unsuitable for long-working-distance topography measurement.

Krutz et al. [104] suggested plenoptic cameras for small working distances and the same group tested a commercial plenoptic camera for measurement of Martian rock [18]. The experimental setup had a 160 mm working distance and characterization tests with a textured planar sample produced an RMS depth error of approximately 150 µm. The system laterally resolved approximately 198 µm/px with 50% contrast over the complete 58 mm FOV. Neither magnification changes nor pixel-size changes nor trading lateral for axial resolution enable both depth and lateral resolution to be below 10 µm. A similar ratio of RMS depth error (approximately 440 µm) to working distance (approximately 450 mm) was published by a commercial supplier of plenoptic cameras in 2016 [105]. These findings suggest that plenoptic cameras are not suitable for front-mounted or bottom-mounted applications in this work.

### Appendix E.3. Structure from Motion (SfM)

The rover *Perseverance* uses this approach, combining multiple cameras and motion, to generate 3D measurements of its environment [9]. The pose of the camera is calculated from homologous points in the images. Single-camera SfM with microscopic resolution on a small rover requires tip/tilt scanning. This is because viewing direction as well as camera translations are necessary to generate homologous points with large triangulation angles and small fields of view. To increase homologous point detection, multiple images are taken at smaller increments, with each image requiring rover motion, scanning, refocusing and focal stacking, leading to long measurement times. Active illumination cannot be used to increase sampling density in rover-borne SfM, as projected features will shift with rover motion.

SfM is also limited by Equation (11), and the triangulation angle produced by rover motion could be large. Practically, errors in knowledge of motion, camera calibration and algorithms are more limiting. As a practical example, seven commercial SfM software packages were tested for cultural artifact documentation, where 20 highly resolved (20 µm/pixel or better) images taken of rotating (18° increments) natural stone objects had standard deviations of at least 378 µm [106]. This suggests that the technique is not suitable for submillimeter-scale topographic measurement.

To the authors’ knowledge, the spatial performance testing of SfM on *Perseverance* has not been made public. It is suggested in [9] that the main advantage is the creation of large 3D scenes and scientific interpretation must consider the spatial artifacts generated. The Mastcam-Z lateral resolution [14], being finer than the other external facing cameras [3,107], is a few hundred micrometers, suggesting that depth resolution below a hundred micrometers is theoretically, though not practically, possible for *Perseverance* without the robotic arm.

For SfM with orthogonal viewing directions, the measurement volume is approximately given by the cube of the imager lateral extent of the object field (Δ*x* in Equation (5)). SfM requires the baseline imager and a 1D tilt-scanning mechanism with a large angular range.

### Appendix E.4. Other Precluded Techniques

Time-of-flight techniques were not included due to insufficient depth resolution and the need for high-speed electronics and scanning. Approaches based on transport of intensity equation [108] were not included due to disputed quantitative accuracy. Shape from shadowing without a large controlled range of illumination angles cannot reliably capture arbitrary topographies. Fourier ptychography [109] can measure opaque objects with an imaging camera and a large illumination ring [110], but millimeter-depth-scale objects have not yet been shown in the literature. Real-space ptychography, unlike Fourier ptychography, requires mechanical translation of the object. For these two reasons, ptychography is ill suited to this application.

Another binary fringe projection technique is laser triangulation profilometry, where a scanned laser spot or line replaced the stationary pattern. The disparity measurement uncertainty *s_x_* in Equation (11) may be reduced below 1 pixel for some surface types if the centroid of the projection spot/line can be calculated. Although it is a simple and robust technique, unlike binary FPP it requires a one- or two-axis scanner, and so is inferior for this application.

Multiview confocal laser scanning microscopy [51] use pinhole arrays to simultaneously capture depth-sectioned images without lateral scanning. However, illumination efficiency is poor and dithering is needed to improve poor sampling density. The Raman/LIBS laser cannot be divided by such a pinhole array and retain sufficient intensity for spectroscopic excitation. Finally, the dithering mechanism and separation of multiview CLSM from the spectrometer optical path add mass/volume.

Optical scanning holography [111] is a speckle-free holography technique using a coherent source. A heterodyne Fresnel-zone-plate illumination pattern is laterally scanned across the object and detected at a point detector. The approach was precluded here due to the necessary 2D scanning system and multiple lasers required to achieve multispectral topography measurement.

In in situ astrogeology, measurement certainty is critical, as repeated measurements are impossible. Machine-learning methods contain unpredictable and unknowable errors when combined with samples unknowable a priori, so they are excluded from the scope of this paper.
